# Seasonal influence on postoperative hip fracture complications: Retrospective cohort of more than 1000 patients from a tertiary-care university hospital

**DOI:** 10.1016/j.amsu.2020.06.022

**Published:** 2020-06-20

**Authors:** Obada Hasan, Mashal Amin, Fatima Mahmood, Amna Rabbani, Umar Rabbani, Shahryar Noordin

**Affiliations:** a Orthopaedic & Rehabilitation Department, University of Iowa, USA; b Department of Pediatric Medicine, The Aga Khan University Hospital, Pakistan; c The Aga Khan University, Pakistan; d Department of Surgery, Section of Orthopedics, The Aga Khan University Hospital, Pakistan

**Keywords:** Hip fracture, Surgery, Season, Summer, Complication

## Abstract

**Background:**

Hip fractures account for one of the most debilitating conditions affecting the general population amongst the developed and developing nations. Investigators aimed to study the influence of the ongoing season i.e. whether patients operated upon in summer or winter, on post-operative complications occurring within 30 days of surgical procedure for hip fractures at a tertiary care hospital and level 1 trauma center.

**Materials and methods:**

Investigators conducted a non-funded, non-commercialized retrospective cohort of 1045 patients with hip fractures managed surgically. Primary exposure was surgical procedures undertaken during the summer months (April–September). Our primary outcome was determining post-operative complication rates from January 2010 to December 2018 and evaluating impact of the season through univariate and multivariable regression analyses using Cox Proportional Hazard Algorithm with STATA V15. The work has been reported in line with the STROCSS criteria.

**Results:**

Age, ASA status, type of procedure, mechanism of injury and Charlson Comorbidity Index (CCI) were identified as significant associated factors with postoperative complications after hip fracture surgery. Although overall results revealed a rising rate of complications during the summer season with a relative risk (RR; 95% CI) of 1.1(0.89–1.32), univariate and multivariable analysis did not show any significant correlations.

**Conclusions:**

The results of this study did not demonstrate a significant seasonal variability in the rate of postoperative complications for hip fracture patients operated upon during the hot months of summer. More research is required to analyze postoperative complications in order to optimize patients’ outcomes.

## Highlights

• The number of hip fractures has been observed to double as the population ages.
• Surgical management is the gold standard and most effective management option.
• Operating patients during summer is postulated to carry higher risk of postoperative complications.
• However, this higher risk was statistically insignificant in the current study.

## 1 Introduction

Hip fractures account for one of the most debilitating conditions affecting the population amongst the developed and developing nations. It not only poses a direct economic burden on the medical system but also affects patients in terms of social and psychiatric domains. The number of hip fractures has been observed to double as the population ages [1]. Considering the medical advancements and increased awareness regarding development of a healthy lifestyle, it is no surprise that the life expectancy is increasing, consequently increasing the prevalence of hip fracture.

Management of hip fractures is a common orthopedic procedure which holds great societal and personal benefits for these patients [2]. However, some patients develop complications post-operatively leading to longer hospital stays and readmissions with the associated financial burden. Therefore, it is important to identify factors that increase the risk of complications which can be mitigated to enhance patient outcomes. Some of these factors include surgeon expertise, hospital type, age and comorbidities of the patient, type of anesthesia, day of the week and time lapse between admission and procedure amongst several others [[3], [4], [5]]. Another important factor coming to light is the influence of the ongoing season on rate of complication with multiple studies presenting data showing significant rise during summer months [6,7]. Variation in season and surrounding are known to have a profound effect on one's mood, behavior and lifestyle. This is also having an effect on patient's medical response and studies have shown that few people turn up for appointments during winters. Similarly, in summers we witness increased Vitamin D levels and decreased survival of microorganisms [7]. Another interesting phenomenon arising during summers is the July effect [8], which is an increase in morbidity and mortality observed in patients admitted between July to August which coincides with admission of new interns and residents. Most of the data on post-operative complications following hip surgery has been collected from developed Western countries which cannot be extrapolated to developing countries due to a major difference in overall climate, temperature, humidity and precipitation levels. Therefore, our study reviews the impact of the equatorial climate on complications occurring after surgical management of hip fractures. Changes in weather conditions can have a huge impact on the outcomes of surgical procedures depending on the time of the year when the surgery was performed. However, very few studies have been conducted worldwide to study the differences in the 30-day postoperative outcomes of hip surgeries based on the seasons (summer versus winter season), and even fewer studies have been done in Pakistan. Therefore, we conducted a retrospective study to study the effect of seasonal variations on post-operative outcomes of hip surgeries.

## 2 Methodology

### 2.1 Study design and study setting

This is a single-hospital based case control study conducted at the section of orthopaedics in the department of surgery at the country's largest private referral tertiary care university hospital and a level-1 trauma center. This University Hospital is a Joint Commission International (JCI) accredited center located in the world's seventh largest metropolitan city. Investigators obtained University Ethical Review Committee approval and registered the study at clinicaltrials.gov. The research team included specialists in the fields of orthopaedic surgery, epidemiology and biostatistics. Data collectors were interns, who were graduates of the same institute and trained in data collection process and management. Protocol was developed before and available with the corresponding author on request.

### 2.2 Patients’ selection and eligibility criteria

A retrospective data collection of patients was done, who were admitted at the University Hospital from January 2010 to December 2018. All patients, regardless of gender, comorbidities and age, who had hip fractures and managed surgically, were included in the study. Patients with incomplete data or missing information in either the primary exposure or the outcome of interest were excluded from the study. Patients with pathological fracture, open fractures, polytrauma or revision surgeries were also excluded. A total of 1045 patients were included in the study and the data extracted from their medical records were analyzed. Results were reported as per STROCSS criteria [14].

All orthopedic surgical procedures were performed by Orthopaedic surgeons who were credentialed by the University Hospital Credentialing Committee to do the procedures. All patients underwent a standardized preoperative antibiotic protocol which was started an hour before surgery and continued for the next 24 h. Surgical site was prepped using pyodine soap/solution before draping. All patients underwent a standard postoperative protocol in the inpatient ward including mobilization and physiotherapy based on the fracture type and surgical procedure performed as per the surgeon's instructions. This was followed up by home physiotherapy sessions as per patient need. Patients were closely followed up after discharge for 30 days to monitor for any postoperative complications.

### 2.3 Data management, primary exposure, outcome and analysis plan

Medical records were reviewed and data were entered in statistical software for analysis. We recorded demographic data (age, gender, body mass index, comorbidities, American Society of Anesthesiologists grade, Charlson Comorbidity Index (CCI), date of surgery, mechanism of injury, walking status prior to surgery) and clinical data (type of surgery, type of procedure, duration of surgery, postoperative intensive care unit stay, length of stay and postoperative complications).

Primary exposure was if patient was operated upon during summer season. Month of surgery was extracted from the confirmed dates in our system and was appropriately set as a categorical variable with 1, January; 2, February; 3, March and so on. We then decided to divide the months into 2 seasons of six months, summer months (April–September) and winter months (October–March). Primary outcome of interest was postoperative complications (in-hospital and 30 days postoperative).

For descriptive purpose, Pearson chi-square test was used for the analysis of categorical variables and two samples Independent T test was used for analyzing continuous variables. Following crude analysis, Cox Proportional hazard regression was also carried out to adjust for potential confounders or interactions and significant variables associated with the binary outcome variable i.e. post-operative complication. For all univariate analysis, *p* value of less than and equal to 0.25 was considered significant and for all multivariable analysis *p* value of less than and equal to 0.05 was considered significant. STATA V15 was used for analysis.

## 3 Results

**Fig. 1:**
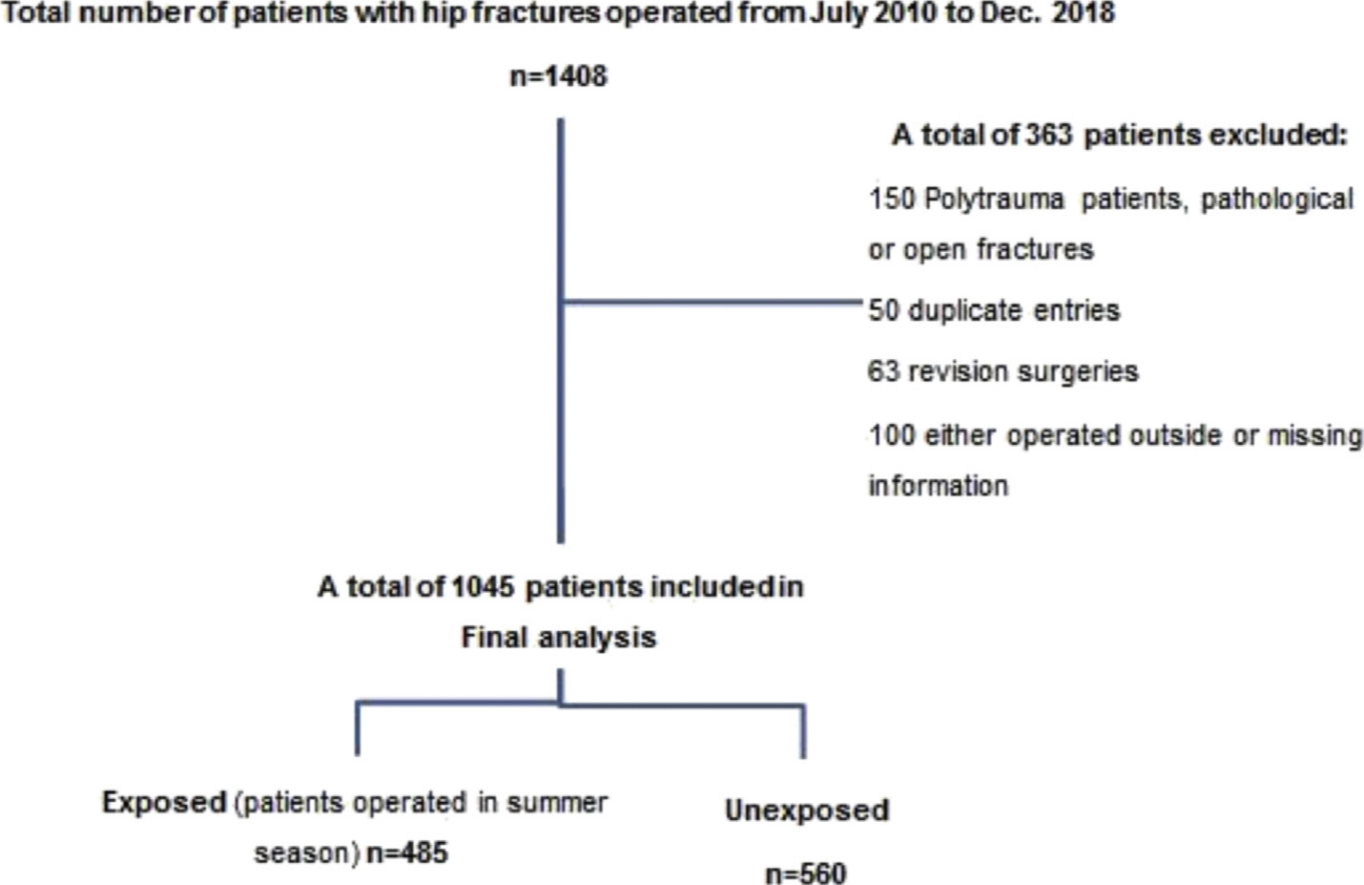
Flow chart showing patients' screening and selection.

After screening, a total of 1045 patients who underwent hip fracture surgeries were included in the final analysis (Flow chart in Fig. 1). Of these, 485 (46%) patients underwent their hip fracture procedure during summer months (April–September) and 560 (54%) patients underwent surgery during winter months (October–March). Of the total 485 patients in exposed group, 203 (42%) patients had post-operative complications while 215 patients (38%) of the unexposed group had complications. However, result was statistically insignificant.

Mean ± SD of age of patients in both groups was almost equal at about 67 ± 1 year. Mean duration of surgery in the summer months was slightly greater than the mean duration of surgery in winter months, i.e. 106 and 101 min, respectively. Men made 47% of patients in the exposed group while 53% men had their surgery in the winter months. Body mass index of the patients who had surgeries in the summer and winter months were, 42.5% and 57.5%; 47% and 53% were normal weight, 47% and 53% were overweight, 43% and 57% were class I obese, 47% and 53% were class II obese and 66.7% and 33.3% were class III obese, respectively. Both groups were comparable with insignificant differences except for mechanism of injury and type of anesthesia (Table 1).

**Table 1:** Demographic and clinical characteristics of exposed (operated in summer) and un-exposed groups.

Variables	Exposed	Unexposed	p value^a^ (0.05)	Variables	Exposed		Unexposed		p value (0.05)^a^
**Surgery duration (Minutes) Mean±SD**	106±(2)	101± [2]	0.05						
	**n(%) n(%)**				**n(%) n(%)**				
**Gender**			0.57	**Procedure**^b^				0.06	
Men	218 (47%)	242 (52.6%)		DHS	225 (43%)	296 (57%)			
Women	267 (45.6%)	318 (54.4%)		Bipolar Hemi.^b^	42 (44%)	54 (56%)			
**ASA Status**			0.06	Monopolar Hemi.	60 (44%)	76 (56%)			
I	51 (59%)	35 (41%)		THR	67 (54)	56 (46%)			
II	202 (47%)	229 (53%)		Nailing	30 (49%)	31 (51%)			
III	206 (44%)	260 (56%)		Others	61 (56%)	47 (44%)			
IV	26 (42%)	36 (58%)							
**Anesthesia Type**			0.05	**Injury Mechanism**				0.02	
GA	366 (48%)	393 (52%)		Ground Level Fall	396 (45%)	485 (55%)			
Regional	119 (42%)	167 (58%)		High energy	89 (54%)	75 (46%)			

a Proportions in the two groups are compared using Chi-square test and independent *t*-test. P value of ≤0.05 is significant.

b Procedures: Hemi. = Hemiarthroplasty, Others = Canulated screws/plates.

### 3.1 Univariate analysis

On univariate level using the cox proportional hazard analysis as presented in Table 2, we observed that season had a relative risk of 1.1 with CI (0.89–1.32). Age, ASA status, type of procedure, mechanism of injury and CCI were the only significant variables with a *p* value of less than or equal to 0.25. Patients with advanced ASA level had a risk of having post-operative complication 2.08 times higher as compare to a patient with ASA level 1–2 [RR = 2.08, CI = (1.21–3.57)]. Patients with severe comorbids in CCI were found to have 1.83 time higher risk of developing a post-operative complication as compare to a normal CCI [RR = 1.83, CI = (1.31–3.32) and *p* value of <0.01].

**Table 2:** Factors associated with postoperative complications after hip fracture surgery at the Univariate level using Cox Proportional Algorithm Analysis reporting RR with 95% Confidence Interval.

Variables	RR (95% CI)	P value (0.25)
**Primary Exposure**	1.09 (0.89–1.32)	0.38
**Age(years)**	1.01 (1.00–1.02)	**<0.01**
**ASA Status**		**0.02**
I	1	
II	1.45 (0.93–2.27)	
III	1.70 (1.09–2.64)	
IV	2.08 (1.21–3.57)	
**Procedure Type**		0.19
Elective	1	
Emergency	0.88 (0.72–1.07)	
**Injury Mechanism**		**0.01**
Ground Level Fall	1	
High Energy	0.69 (0.52–0.94)	
**CCI**^a^		**<0.01**
None	1	
Mild	1.24 (0.71–2.27)	
Moderate	1.60 (1.09–2.87)	
Severe	1.83 (1.31–3.32)	

a CCI: Charlson Comorbidity Index.

### 3.2 Multivariate analysis

Patients who underwent their surgeries during the summer months had 10% higher risk of developing post-operative complications as compared to patients who had their surgeries during the winter months [aRR = 1.10, CI = (0.99–1.33)] after adjusting for all other independent variables. However, this was statistically insignificant (*p* value 0.33). Patients who had CCI as mild, moderate and severe comorbids had a higher risk (ranging from 23% to 82% respectively) of developing a post-operative complication as compare to patients who did not have comorbids, after adjusting for all other variables [aRR = 1.23, CI (0.72–2.26)], [aRR = 1.60, CI = (0.99–2.60)] and [aRR = 1.82, CI = (1.14–2.91)] respectively (Table 3).

**Table 3:** Final model after multivariable analysis for factors associated with postoperative complications after hip fracture surgery.

Variables	aRR^a^ (95% CI)	p Value
**Primary Exposure**	1.10 (0.99–1.33)	0.33
**CCI**		
Mild	1.23 (0.72–2.26)	**<0.01**
Moderate	1.60 (0.99–2.60)	
Severe	1.82 (1.14–2.91)	

a **aOR: Adjusted Relative Risk. C.I.: 95% Confidence Interval. P value of ≤ 0.05 is significant**.

## 4 Discussion

Our study revealed that patients who underwent their hip fracture surgeries during the summer months have a 10% higher risk of developing postoperative complications as compared to patients who had their surgeries during the winter months [aRR = 1.10, CI = (0.99–1.33)], after adjusting for all other independent variables. But even though we did report differences in the incidence rates of 30-day post op complications after hip surgery between the different seasons, our results were statistically insignificant. However, we did find a significant effect of age, ASA status, type of procedure, mechanism of injury and CCI. Moreover, after adjusting for all other variables, we found that patients with mild, moderate and severe co-morbids had a higher risk (ranging from 23% to 82% respectively) of developing a post-operative complication as compare to patients who did not have any co-morbids.

This was in agreement with a previous study done by Malik et al. [9], who reported that a preoperative comorbidity of chronic kidney disease (OR, 3.27; 95% CI, 1.15 to 9.28) was associated with a higher odds of developing inpatient complications. He also concluded that patients who underwent surgery during May–August were twice more likely to suffer from postoperative complications than those who had surgery in the winter months. However, there was no increase in risk for SSIs and prolonged length of stay during the summer months(9).Another study also reported the effect of chronic kidney disease comorbidity on post-operative complications. Ackland et al. [10] found out that most of their patients with CKD undergoing surgery had a longer postoperative hospital stay, had a greater risk of morbidity and took longer to get better post operatively. Consistent with the above studies, Anthony et al. also reported that patients with the diagnosis of diabetes, hypertension, and obesity were all at higher risk for SSIs and complications following both TKAs and THAs [11].

Other investigators have also studied the relationship between seasons and postoperative infections. The results of Mitchell Ng et al. [12] were in concordance with our study, reporting no significant correlation between seasonal variation and 30-day complication rates following total hip arthroplasty surgery.

In contrast to our study, other authors have reported a significant association between time of year and postoperative complications. In a study conducted to find the association between the time of the year and post op outcomes of total knee arthroplasty, Sodhi et al. [8] reported significant differences in the incidence of superficial infection, pneumonia, and blood transfusion at different times of the year in both univariate and multivariate analyses (P < .05). These results are similar to the work of Anthony et al. [11], who reported that incidence of surgical site infections (SSIs) during summer among patients undergoing TKA and THA is higher (being highest in June) compared to incidence of SSIs in winter (being lowest in December). A significant difference in the incidence of acute postoperative infections by season in the Western part of the United States was also reported by Rosas et al. [13]. The surgeries performed in the summer had the greatest incidence of post op infections (1.59%), compared to all other seasons (P < 0.001 for all). However, no difference in postoperative infections was found when stratified by season in the other regions of the United States. While analyzing these studies, one needs to take into account the geographical variation as our study was conducted in Pakistan and the above mentioned studies were conducted in the United States. Moreover, we also need to consider the ‘July effect’ (hypothetical increase in morbidity thought to be associated with the training of new residents during the first portion of the academic year) [12], present in the hospitals in the United States which could be a major confounder, as this doesn't happen in Pakistan since the induction of new interns and residents in our hospitals starts in January.

## 5 Strengths

Our study is a cohort design with sufficient number of cases and follow-up as per the objectives and conducted by an experienced team in surgical field, clinical research and data management. That reduces the selection or information bias. Clinical outcomes reported are of at most importance to surgeons and patients as well.

## 6 Caveats and future study recommendations

Retrospective design with chart review is one of the main limitations of our study as many files with missing information were excluded from the study. Our data set did not include patients who were managed non-operatively. Furthermore, the surgeries were performed by multiple surgeons, even though they were all experienced surgeons, it could still be a source of a risk of bias as different surgeons have different preferences with respect to surgical approaches. Moreover, although categorizing the age into groups was not significantly associated with the primary exposure or outcome, including all adult patients in the sample can increases the external validity but at the expense of increasing the chance of information bias. Another limitation was limiting the post-operative complication period to 30 days; if we had been able to extend this period, we might have been able to see a stronger association. Further research, including prospective designs, should be done to study the differences between different procedures and to establish evidence-based care guidelines.

## 7 Conclusion

Our study showed that seasonality does not influence the rate of postoperative infections following hip fracture surgeries. More research is needed to explore the link between time of the year and post-operative complications so that morbidity and mortality following hip surgery and outcomes can be further enhanced.

## Level of evidence

Level II, comparative study; retrospective cohort.

## Disclaimer

None.

## Financial support and sponsorship

Nil.

## Provenance and peer review

Not commissioned, externally peer reviewed.

## CRediT authorship contribution statement

**Obada Hasan:** Conceptualization, Methodology, Validation, Investigation, Writing - review & editing. **Mashal Amin:** Methodology, Investigation, Writing - review & editing. **Fatima Mahmood:** Investigation, Methodology. **Amna Rabbani:** Methodology, Investigation, Writing - review & editing. **Umar Rabbani:** Methodology, Investigation, Writing - review & editing. **Shahryar Noordin:** Conceptualization, Investigation, Writing - review & editing, Supervision.

## Declaration of competing interest

There are no conflicts of interest.
